# The coronavirus disease 2019 infodemic: a concept analysis

**DOI:** 10.3389/fpubh.2024.1362009

**Published:** 2024-04-25

**Authors:** Sujin Choi

**Affiliations:** Department of Nursing, College of Medicine, Soonchunhyang University, Asan-si, Republic of Korea

**Keywords:** infodemic, overload, asymmetry, reproduction, dissemination

## Abstract

**Aim:**

This study aimed to analyze the coronavirus disease 2019 (COVID-19) infodemic phenomenon in the medical field, providing essential data to help healthcare professionals understand it.

**Methods:**

This study utilized a hybrid model for concept analysis. In the theoretical phase (first phase), a literature review was conducted using ScienceDirect, PubMed, CINAHL, ProQuest, Scopus, Web of Science, DBpia, RISS, and KISS. Semi-structured interviews, involving eight physicians and six nurses, were used in the fieldwork phase (second phase). In the final analysis phase (third phase), the results of the preceding phases were combined.

**Results:**

Based on the findings of these phases, the COVID-19 infodemic can be defined as “the phenomenon of information flood, reproduction, dissemination, and asymmetry, which occurred during the pandemic through social networks among the public lacking essential knowledge of infectious disease, and is associated with negative and positive effects.”

**Conclusion:**

Our findings can help the Ministry of Health and Welfare and healthcare professionals to understand the phenomenon of the infodemic and prepare necessary strategies and education programs for the public. Therefore, the provision of basic data is important for developing influential roles for healthcare professionals during infectious disease outbreaks.

## 1 Introduction

According to the World Health Organisation (WHO), the information tsunami during the coronavirus disease 2019 (COVID-19) pandemic resulted in the generation of fake news that lacked scientific evidence and conveyed misunderstandings and misinformation about health (1). After WHO declared COVID-19 as a pandemic in March 2020 (1), an accompanying phenomenon called the “information pandemic” emerged, which refers to the rapid spread of misinformation or fake news through social media platforms and other mass media (2). Previous research has indicated that the information pandemic during the COVID-19 period which has called “COVID-19 infodemic” caused an invisible disaster with serious and widespread harmful effects (3, 4). Additionally, WHO defined an infodemic as a state in which correct and incorrect health information is mixed and proclaimed their combat against the infodemic (1).

Moon and Lee (5) analyzed the 200 most-viewed Korean YouTube videos about the COVID-19 virus in 2020, and identified that YouTube users created most videos, and that 37.13% of the videos contained incorrect information, with each video reflecting up to 68.09% of misinformation. Examples of misinformation included that boiling water, snake oil, silver, and burning incense could treat COVID-19 (6), and conspiracy theories suggesting that the government put microchips in the COVID-19 vaccine to track citizens (7).

The infodemic phenomenon negatively affected individuals and the approaches of healthcare professionals and government policies in managing COVID-19. The infodemic during the COVID-19 pandemic also worsened the emotional problems of the public (8). A study conducted in China revealed that frequent exposure to social media containing COVID-19-related content increased depression and the prevalence of hyper-anxiety (9). The phenomenon of people trusting misinformation more than medical staff was also reported (10). Owing to the spread of misleading news, governments worldwide faced challenges in preventing and managing infectious diseases, as the public exhibited reluctance to follow COVID-19 guidelines during the pandemic (11, 12).

While, studies on the causes (13, 14), impacts (8, 14–16), and preventive strategies (14, 17) of the COVID-19 infodemic have been actively conducted, no research has identified to reveal the concept of the COVID-19 infodemic. Conducting a concept analysis enhances the practicality of the concept by providing a clear and transparent definition, thus serving as a foundation for planning, implementing, and assessing the utilization of the concept (18). Pope et al. (19) conducted a concept analysis study on the concept of “health misinformation” during the COVID-19 pandemic, but did not include correct health information. Therefore, it is necessary to conduct analytical research on the entire concept of infodemic, including correct information, as WHO (1) suggested.

Additionally, the need to identify the concept of the COVID-19 infodemic through a concept analysis study in medical settings has been raised. This is because healthcare professionals in medical settings have been at front-line of COVID-19 patients during the pandemic. During the COVID-19 pandemic, healthcare professionals communicated with each other constantly to stay informed amidst the flood of information and make medical decisions (20). However, there is no clear and concise concept of COVID-19 infodemic which is necessary for them to strategically respond to infodemic for a future pandemic. Thus, this study aimed to analyze the concept of the COVID-19 infodemic through identifying its antecedents, attributes, and consequences in the medical setting, providing basic data to help healthcare professionals understand the phenomenon of the COVID-19 infodemic.

## 2 Methods

This study analyzed the concept of the COVID-19 infodemic, targeting physicians and nurses working in medical settings, using a hybrid model. The hybrid model can clarify concepts and understand them in a situational context (21). Concept analysis through a hybrid model combines inductive and deductive analysis approaches and is used to specify concepts because it can subdivide widely applied concepts (18). The hybrid model is based on a literature review and individual interviews; thus, it can provide detailed data and clear analysis findings about concepts depending on context and situation (22). The hybrid model comprises theoretical, fieldwork, and final analysis phases (21).

### 2.1 The theoretical phase

**Figure 1:**
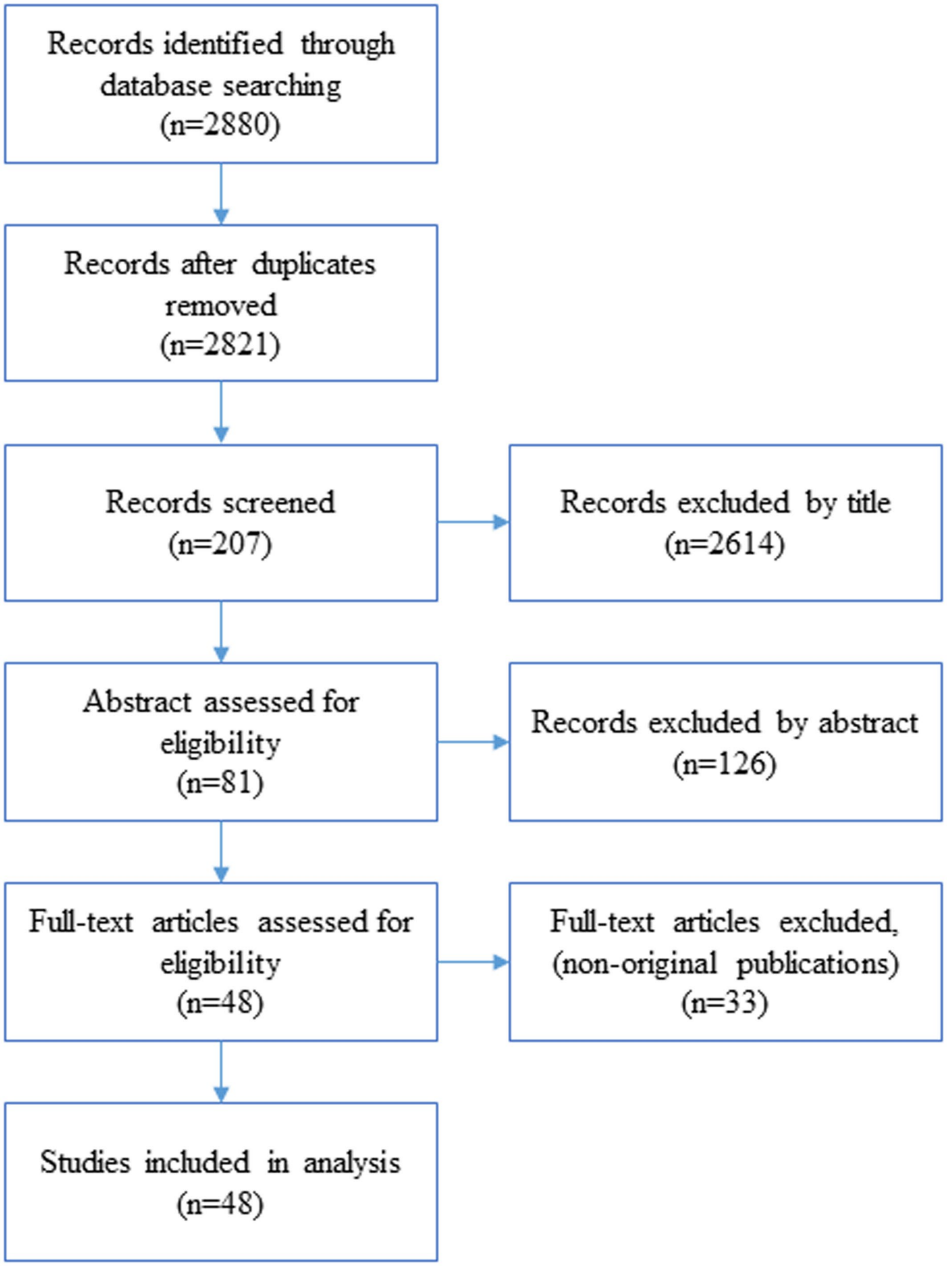
Flow diagram of the selection process.

A literature review was conducted on the infodemic in nursing and healthcare. The literature search included papers published from January 2020 to September 2023 in domestic and international databases such as ScienceDirect, PubMed, CINAHL, ProQuest, Scopus, Web of Science, DBpia, RISS, and KISS. Search terms included “infodemic,” “misinformation,” “information,” “health information,” and “COVID*.” The search strategy incorporated “COVID*” and combined the remaining search terms. The inclusion criteria for papers in the analysis were: (a) inclusion of keywords in the text, (b) publication in English and Korean, (c) availability of full text, and (d) peer-reviewed articles. Editorials, conference discussions, and posters were excluded. Figure 1 illustrates the process of selected studies. A total of 48 eligible articles were included in the study. Following data collection, the content of the selected studies was analyzed, and a detailed definition of the COVID-19 infodemic, along with its antecedents, characteristics, and consequences, was derived.

### 2.2 The fieldwork phase

In this phase, a qualitative study was conducted to explore the first-hand experiences of participants. Eight physicians and six registered nurses were interviewed about the COVID-19 pandemic, its characteristics, antecedents, and consequences. Convenient sampling was employed, ensuring maximum variation in participants’ age, gender, work experience, and healthcare institutions (Table 1). The mean age of the participants was 32 ± 5.3 years.

**Table 1:** Participant demographics (*n* = 14).

Characteristics		n (%)
Age range (years)	20 ~ 29	7
	30 ~ 39	5
	40 ~ 49	2
Gender	Female	9
	Male	5
Occupation	Physician	8
	Registered nurse	6
Education background	Undergraduate	13
	Graduate	1
Work experience (years)	1 ~ 3	5
	3 ~ 6	4
	6 ~ 9	4
	10~	1

Interviews were conducted to explore the experiences of healthcare professionals in medical settings until theoretical data saturation was reached (23). The researcher directly conducted the interviews. The interview questions were: (a) Please tell me about an experience in which patients asked questions about COVID-19 during the COVID-19 pandemic, or an experience in which patients believed nonsensical knowledge; (b) Why has so much information (including misinformation) emerged about COVID-19? What do you think as a healthcare professional; (c) As healthcare professionals, why do you think the public accepts nonsensical knowledge about COVID-19; and (d) Please tell me about any experiences you remember about how patients were later affected positively or negatively by information. The interviews lasted an average of 30 min, and all interviews were recorded using a digital recorder after obtaining consent from the participants. Data analysis was conducted immediately after data collection using Graneheim and Lundman’s content analysis method (24). Each interview data was transcribed into a transcript, read several times to identify keywords and meaning units, and coded to recognize them. Similar codes were grouped to derive themes. The researcher, having extensive experience in qualitative research, wrote reflection notes on the researcher’s biases and preconceptions before the interview and utilized them in data analysis to improve the quality of the research results and avoid possible bias. Furthermore, the researcher employed a rigorous process to cross-verify responses from participants whose interview data carried ambiguous meanings. Through this iterative approach, data saturation was attained.

### 2.3 Final analysis phase

The results from the preceding two phases were combined. Subcategories were constructed by comparing and merging the codes extracted from the two phases. Finally, attributes, antecedent factors, and consequent factors were identified to provide a comprehensive definition of the concept.

## 3 Results

### 3.1 Findings of the theoretical phase

#### 3.1.1 Definition of an infodemic

At the beginning of the COVID-19 pandemic, WHO announced the term and defined an “infodemic” as “too much information, including false or misleading information, in digital and physical environments during a disease outbreak” (1). This term has been used to describe the rapid spread of information, both online and offline (25), covering various aspects such as the virus, disease, treatment, standard operating procedures, lockdowns, and vaccines (26). Before the COVID-19 pandemic, such unverified and inaccurate information encompassed misinformation, disinformation, and malinformation (27).

#### 3.1.2 Antecedents of the infodemic

The antecedents of the infodemic were categorized into environment-related and public-related.

##### 3.1.2.1 Environment-related

A pandemic is defined as “an epidemic occurring worldwide or over a wide area, crossing international boundaries and usually affecting numerous people” (28). The pandemic resulted in an infodemic (26). Research has revealed that misinformation can foster an atmosphere of panic and discrimination in pandemics (29). The dissemination and consumption of information have spiked since the COVID-19 pandemic (30). At the onset of the pandemic, consumption of news among the public increased by 62% (31), with many being exposed to significant amounts of misinformation and fake news while seeking information related to COVID-19 pandemic (32, 33). Pandemics have resulted in infodemic even before COVID-19. For example, a rumor claiming that lack of iodine caused severe acute respiratory syndrome led to panic buying of salt during that pandemic in China (34).

Social media affects infodemic. A rapid integrative review study on infodemic during the COVID-19 pandemic reported social media as a direct source of quickly disseminating misinformation (4, 35). Another systemic review on health misinformation on social media identified high levels of misinformation on vaccines and disease on Twitter (36). Social media and private unfiltered networks such as WhatsApp, Facebook, Twitter, YouTube, and TikTok spread information much faster than the virus (37). A retrospective analysis of the COVID-19 infodemic in Saudi Arabia identified three sources of rumors social paths (through talking with friends and family), (2) traditional media such as television and newspapers, and (3) social media platforms such as Twitter and Facebook which were reported as the most common source of rumors, as these platforms are now the go-to media for information (25). Additionally, a study analyzing data on the COVID-19 social media infodemic reported that information from reliable and questionable sources does not present different spreading patterns (4).

##### 3.1.2.2 Public-related

People with a low level of knowledge about COVID-19, low health/media literacy (17), and low trust in government/news media, particularly those with lower education, males, and younger individuals (26), tend to be more susceptible to the infodemic. Another study revealed that people with high levels of health literacy experienced difficulties dealing with the infodemic during the COVID-19 pandemic (38). This contrasts with research findings suggesting that people with low awareness (26) are more likely to be exposed to infodemic.

#### 3.1.3 Characteristics of the infodemic

The characteristics of the infodemic were identified as quantitative volume of information and qualitative pattern of information.

##### 3.1.3.1 Quantitative volume of information

A survey among healthcare professionals in India reported that 67% of respondents either agreed or strongly agreed about information overload (39). The types of information include unreliable information, rumors, and gossip (39), and false news, conspiracy theories, magical cures, and racist news (35, 40). Misinformation and disinformation about the virus, its origin, the vaccines, and potential treatment proliferated throughout the COVID-19 pandemic (41). Compared with that a decade ago, access to the internet and smartphones, as well as the availability of laptops at much cheaper rates, has facilitated the collection and real-time sharing of data, collaboration across different continents, live video conferences to share experiences, uploading of educational videos, and the accessibility of scientific information as soon as it becomes available (40).

##### 3.1.3.2 Qualitative pattern of information

Wardle and Derakhshan discussed the three elements involved in the creation, production, distribution, and reproduction of misinformation (42). The created information is reproduced through the combination of social media and personal experiences. Social media users interpret the reproductive information and distribute it, with many regular users contributing to most retweets of content sourced from fake news websites (43). WHO also detected the production of fake news from the tsunami of information during the COVID-19 pandemic (1). A survey among healthcare professionals in India reported that 75% of respondents either agreed or strongly agreed about inaccurate information. Fifty percent of the respondents agreed or strongly agreed that differentiating correct from incorrect information is challenging (39).

Studies have documented the global spread of information and misinformation in the context of COVID-19 (39). The term “infodemic” has been used to describe the rapid spread and sharing of information (39, 40, 44). A rapid review study on misinformation during public health emergencies due to pandemics identified the sources of information from social media, friends and family, healthcare providers, religious leaders, and word of mouth (35). Some researchers evaluated the spreading pattern of news on COVID-19. Cinelli et al. revealed that the spread of information is motivated by the interaction paradigm set by the specific social media platforms and/or by the interaction patterns of users engaged in the topic (4). Pennycook et al. discovered that people shared false news about COVID-19 partially because they did not adequately consider the accuracy of the content before deciding to share (45).

#### 3.1.4 Consequences of the infodemic

##### 3.1.4.1 Impact on wellbeing

An infodemic causes confusion, panic attacks (29, 46), and fear and anxiety among citizens (37, 44). The fear of the virus created by social media is more contagious to the general population than COVID-19 itself (37). For example, a man in India who was hospitalized for treatment by healthcare professionals committed suicide because of unclear information (47). Vaccination hesitance, which is the refusal of vaccines when access is not a limiting factor, has also been reported (48). In addition, information avoidance was reported. An overabundance of COVID-19 information can harm mental wellbeing and lead to a discontinuation of information-seeking behavior, as people deliberately avoid information that threatens their wellbeing (49).

##### 3.1.4.2 Impact on healthcare policy

An infodemic triggers discrimination and stigma of disease and hinders the rapid response policies of health officials and policymakers (50). Infodemic can cause confusion and risk-taking behavior, which can harm an individual’s health, and cause mistrust in healthcare authorities (51), lengthening the outbreak (52). An infodemic makes it challenging for the public to comply with public health measures, as it can debilitate individuals’ ability to distinguish mis- and disinformation from fact and cause false perceptions of true risk, including a higher perceived risk and a false sense of safety (38, 53).

### 3.2 Findings of the fieldwork phase

In this phase, 185 primary codes were generated and grouped into three main categories: dimensions, antecedents, and consequences of the infodemic (Table 2).

**Table 2:** Hybrid data analysis in COVID-19 infodemic.

The phase of the study	Codes	Subcategories	Categories
Theoretical phase	Information overload	Quantitative volume of information	Characteristics of COVID-19 infodemic
	Reproduction of information	Qualitative pattern of information	
	Rapid spread of information		
	The pandemic	Environment-related	Antecedents of COVID-19 infodemic
	The development of social network services (SNS) The use of SNS		
	Being unprepared to disease outbreak among the public	Public-related	
	Anxiety, fear, suicide, vaccination hesitance, information avoidance	Impact on wellbeing	Consequences of COVID-19 infodemic
	Not responding to health policies Mistrust in healthcare authorities	Impact on healthcare policy	
Field work phase	Information flood	Quantitative volume of information	Characteristics of COVID-19 infodemic
	Reproduction of information	Qualitative pattern of information	
	Dissemination of information		
	Asymmetry of information		
	Usage of social network services Limited access to healthcare professional for home-based treatment	Environment-related	Antecedents of COVID-19 infodemic
	Absence of essential understanding on infectious disease	Public-related	
	An increase in interests in the COVID-19 virus among the public Practicing preventive measures cautiously	Positive impacts	Consequences of COVID-19 infodemic
	A decrease in trust in healthcare professionals Creation of anxiety and confusion among patients	Negative impacts	

#### 3.2.1 Characteristics of infodemic

The subcategories of the characteristics of the COVID-19 infodemic were identified, consistent with the findings of the theoretical work. A code for the subcategory “asymmetry of information” under the category of “qualitative pattern of information” was additionally derived.

##### 3.2.1.1 Quantitative volume of information

Most participants recalled the COVID-19 pandemic period, identifying an overload of unnecessary information, such as all the movement routes of people with the COVID-19 virus, newsletters regarding treatments from reporters who did not fully understand the medical information, and information on late complications of the COVID-19 virus (Participants 3, 6 and 12). They mentioned that the quantity of other types of information was overwhelming compared with the information provided by healthcare professionals (Participant 5). Furthermore, much information was available but tended to be repetitive (Participant 9).

*As you know, they now announce the number of confirmed cases every day, and we receive several messages. It is so overwhelming to the point that it feels like a trauma, with so much information. At first, when there were not many initial confirmed cases, they disclosed all the movement routes* (Participant 12).

##### 3.2.1.2 Qualitative pattern of information

Most participants highlighted that the public reproduced information. The reasons for the reproduction of information included a lack of basic understanding of medical articles, purposefully creating provocative news to gain more “likes,” and political motives (criticizing the current government’s actions). The phenomenon of information reproduction has become most prominent in the social media space.

*In the case of the media, information is directly linked to profitability based on the number of views, so there have been some indiscriminate articles published, competing with provocative titles and phrases. Someone made claims about things that have not been proven, and when encountering such internet articles, it is easy to be deceived because the internet articles seem more credible than friends or acquaintances* (Participant 6).

Dissemination of information refers to the same characteristic, “rapid spread of information,” drawn from the theoretical work. According to our participants, stopping the dissemination of information through social network services online is impossible. Information spreads within social networking services (SNS) platforms, and family members in a family, coworkers in the workplace, and friends, who also share news they encounter on SNS. This pattern of information dissemination is even faster.

*Nowadays, in a situation where anyone can freely create videos and access information, the creation and dissemination of any information itself has become possible from anyone, anywhere. While it is true that the spread of information has been fast, when I thought about whether it could be controlled, I actually believe that control is impossible* (Participant 8).

Most participants highlighted the asymmetry of information, mostly among healthcare professionals, patients, and healthcare institutions. The amount and quality of information about COVID-19 between healthcare professionals and patients may vary. However, healthcare professionals have expressed deep concerns about the variances in the amount and quality of information among themselves and between primary, secondary, and tertiary healthcare facilities. The deep concern regarding the asymmetry of information mentioned by healthcare professionals indicates their inability, as healthcare providers, to provide accurate information to healthcare recipients consistently.

*There is information asymmetry, and information asymmetry exists between healthcare professionals and patients. I also believe that it exists among healthcare professionals themselves. Additionally, it exists among primary, secondary, and tertiary healthcare institutions* (Participant 3).

#### 3.2.2 Antecedents of the infodemic

Antecedents of the infodemic included environment-related and public-related factors.

##### 3.2.2.1 Environment-related

Most participants mentioned SNS development as an antecedent to the COVID-19 infodemic. Additionally, the characteristics of the COVID-19 virus bolstered the use of SNS among the public. Owing to the high transmission rate and low fatality rate of the COVID-19 virus, most of the patients with mild infection underwent home-based treatment. In the home treatment environment, patients were isolated from other family members and did not have healthcare professionals constantly available, as in the hospital setting. Consequently, patients who underwent home-based treatment relied on social media platforms, which are easily accessible and allow for easy communication to ask questions and seek information.

*It seems that when I was admitted to the hospital because I was sick, there were always healthcare professionals available to ask questions. However, in the case of COVID-19, there are no healthcare professionals available in real-time nearby. As a result, I started searching immediately and accumulated knowledge through platforms like YouTube or Naver blogs* (Participant 6).

##### 3.2.2.2 Public-related

Most participants highlighted the absence of basic knowledge of infectious diseases among the public as a key factor affecting the COVID-19 infodemic. According to them, basic knowledge of infectious disease includes the necessity of vaccination, side effects of vaccines, transmission path, and daily health promotion activities during the pandemic. As such, the public, lacking basic knowledge about infectious diseases, would have had difficulty discerning accurate information from inaccurate information and would have unquestioningly accepted what was said on social media or by acquaintances.

*Now, the general public does not have much medical knowledge and it may not be easy for them to get correct information. Even if they are exposed to something stimulating or incorrect, it may be worse* (Participant 11).

#### 3.2.3 Consequences of the infodemic

The participants stated that the most important consequences of the infodemic were divided into positive and negative effects on the public.

##### 3.2.3.1 Positive impacts

The abundance of information generated interest among the public (Participant 1). With accumulated experience in discerning information (Participant 13), infection prevention measures were practiced cautiously and frequently (Participant 4).

##### 3.2.3.2 Negative impacts

The participants mentioned a decrease in trust in healthcare professionals (Participant 13) and the creation of anxiety and confusion among patients (Participant 14), causing suicide (Participant 1).

### 3.3 Findings of the final analysis phase

A comparison of the findings of the theoretical and fieldwork phases revealed similarities and differences in some subcategories and codes. Most of the literature defined an infodemic as a phenomenon of overloading, reproducing, and spreading information, consistent with those of the fieldwork phase. However, the participants in the fieldwork phase introduced an aspect of the COVID-19 infodemic that was not well-addressed in the literature: the asymmetry of information that occurred between healthcare professionals and healthcare institutions. Based on these findings, the concept of the COVID-19 infodemic can be defined as “the phenomenon of information flood, reproduction, dissemination, and asymmetry that occurred during the pandemic using social networks among the public lacking essential knowledge of infectious diseases. It is associated with negative effects such as confusion, anxiety, fear, vaccination hesitance, information avoidance, low trust in healthcare professionals, and suicide among the public, and positive effects such as generating great interest in infectious diseases, leading to the practice of prevention measure cautiously and the ability to discern information among the public.”

## 4 Discussion

This study analyzed the concept of the COVID-19 infodemic from the perspectives of healthcare professionals. The findings revealed that the COVID-19 infodemic has diverse characteristics and should be considered as a whole, encompassing accurate information and false information.

The antecedents of the COVID-19 infodemic identified in the theoretical work of this study were the pandemic, SNS use, and the public being unprepared for an infectious disease outbreak. The use of SNS was reiterated as an antecedent in the fieldwork phase. This finding was in line with the systematic review of COVID-19 infodemic (14) which identified the causes of COVID-19 infodemic as social media usage. Owing to the development and use of various SNS platforms and the increase in the age range of users, SNS is becoming a means of providing and sharing information further and faster (54). SNS has become a major source of information not only for the general public but also for healthcare providers due to the lack of information caused by COVID-19 co-affected by the novel disease and the initial state of research (55). In the fieldwork phase of this study, healthcare professionals stated that the spread of information through SNS is not preventable. Additionally, the reproduction and dissemination of information, prominently manifested through SNS (36, 37). Thus, exploring effective ways to use SNS to manage the infodemic in the event of an infectious disease outbreak following the COVID-19 virus is necessary (Table 3).

**Table 3:** Categories, subcategories, and codes determined on analytic phase.

Categories	Subcategories	Codes
Characteristics of COVID-19 infodemic	Quantitative volume of information	Information flood
		Information overload
	Qualitative pattern of information	Reproduction of information
		Dissemination of information
		Asymmetry of information
Antecedents of COVID-19 infodemic	Environment related	The pandemic
		Usage of social network services
		Limited access to healthcare professional for home-based treatment
	The public related	Being unprepared to disease outbreak among the public Absence of essential understanding on infectious disease
Consequences of COVID-19 infodemic	Positive impacts	An increase in interests in the COVID-19 virus among the public Practicing preventive measures cautiously
	Negative impacts	A decrease in trust in healthcare professionals Creation of anxiety, confusion, fear, panic attack, information avoidance, vaccination hesitance among patients Not responding to health policies

The fieldwork phase in this study revealed that in South Korea, most cases of mild COVID-19 viral infection symptoms were treated at home. However, accessibility to healthcare professionals was lower at home than in hospitals, and patients, therefore, searched for information about symptoms using easily-accessible SNS. This is because although a call center or telemedicine system has been established for patients receiving treatment at home, its’ healthcare professionals and facility resources are insufficient (56, 57). Furthermore, remote sessions for patient-healthcare professionals cannot fully replicate in-person sessions (17). This highlights the problem of resource support, where home treatment patients were unable to receive information in a timely manner in situations where information was needed. These structural factors should be improved.

Our findings also revealed the absence of an essential understanding of infectious diseases among the public. In the theoretical phase, the public’s low level of education and health literacy (26) were mentioned. Similarly, in the fieldwork phase, the lack of basic knowledge about how the public should behave in an infectious disease epidemic situation was also mentioned. This finding paralleled Pian et al.’s systematic review (14). The public, lacking basic knowledge about infectious diseases, may indiscriminately accept inaccurate information, which may lead to negative health outcomes (26, 48, 49). Gabarron et al.’s systematic review on COVID-19 related misinformation on social media (58) conveyed the same message. To prevent the COVID-19 infodemic, the public needs to have basic knowledge about behavior tips, treatment methods, and infectious diseases (including transmission routes).

In this study, the characteristics such as information overload, reproduction of information, and dissemination of information were identified from both theoretical analysis and fieldwork. Brennen et al. supported these findings and highlighted an intriguing observation from their analysis of fake news instances (59), noting that a small percentage of fake news can reach a large audience due to the amplifying influence of influential figures such as politicians, celebrities, and public figures. Additionally, a WHO technical consultation on infodemic management proposed the necessity of strategic partnerships across various sectors, including social media, technology, academia, and civil society (54). Therefore, securing the involvement of influential healthcare professionals in medical academia is crucial as a countermeasure for managing infodemic from other disease outbreaks.

Asymmetry of information is a characteristic derived from the fieldwork phase. This implies that the public lacks the same information and that disparity exists in the quantity and quality of information among healthcare professionals working in primary, secondary, and tertiary healthcare institutions. A previous study (60) revealed that healthcare professionals are not immune to the impact of infodemic. Doctors, especially primary health care doctors, faced tremendous difficulties as they lacked accurate information about the pathogenesis and treatment of diseases caused by the newly emerged COVID-19. The differences in information among healthcare professionals working in different types of medical institutions may lead to public distrust or hinder legitimate actions of governments requiring public cooperation to control the pandemic (50, 51). This suggests that a channel for providing and rapidly sharing accurate information for healthcare professionals is necessary when responding to an infectious disease pandemic.

The consequences identified in this study, such as confusion, panic attacks, anxiety, fear, and suicide, were consistent in the theoretical and fieldwork phases. Positive effects such as disease prevention, cautious practice of measures, and information discerning were also presented. Besides, many previous studies have addressed the negative consequences of the COVID-19 infodemic such as depression and sleep disorders (61), trust loss, inappropriate protective measures (14), fear, panic, and death from panic purchase (58); however, few studies have suggested positive effects. Such positive consequences were also derived during the fieldwork phase of this study. This may be affected by the data collection which was conducted using a retrospective approach after the end of the COVID-19 pandemic. Moreover, in a study investigating the impacts of misinformation, negative effects were reported as mentioned above. In this study, considering the definition provided by the WHO (1), which encompasses both misinformation and information within the concept of the infodemic, it is inferred that positive effects were also addressed.

Regarding the positive effects on the public (including healthcare professionals) who can discern information, a large amount of information broadens their options, increases interest, and encourages cautious behavior (17). Similarly, a recent study revealed that those who perceived higher risk at the individual and societal levels were more likely to seek information on the Zika virus, demonstrating mobilized preventive intention (62). Systematically investigating and examining the differences in infodemic according to the general characteristics of the public is necessary; however, previous studies have identified that low-educated groups are easily exposed to infodemic (26), leading to information avoidance (49) and vaccination hesitance (48). These findings indicate that in the context of an infectious disease pandemic, providing accurate information to the public and ensuring their understanding of the information can prevent extreme and negative outcomes. The most integral step to minimize the adverse effects of the COVID-19 infodemic is education and the provision of authentic, transparent information from reliable sources (17, 37). A large-scale survey targeting the public is needed to determine what information was and was not needed during the past COVID-19 infodemic. These results should be reflected in preparing measures to enhance the public’s knowledge of infectious diseases.

The limitations of this study deserve attention. This concept analysis only considered articles written in English and Korean. However, it is crucial to incorporate relevant articles in other languages related to the COVID-19 infodemic. Considering that English functions as the international language for scholarly communication and publication, the goal of this study is to encompass the majority of the literature on the COVID-19 infodemic. Furthermore, during the fieldwork phase, interviews were conducted with physicians and nurses who shared their experiences based on the situation in South Korea. Therefore, the findings of this study should be interpreted with caution. Future researches should consider reflecting the perspectives of COVID-19 patients, health officials, and policy makers in terms of infodemic.

## 5 Conclusion

In conclusion, this study revealed that a wide range of characteristics, antecedents, and consequences should be considered in defining the COVID-19 infodemic. The findings contribute to the understanding of the infodemic phenomenon, enabling the Ministry of Health and Welfare and healthcare professionals to formulate necessary strategies and education programs for the public.

Improving access to the right information in a timely manner for patients undergoing home treatment, who often lack access to healthcare professionals, could be addressed by smartly utilizing SNS. Educational programs for the public are crucial for imparting basic knowledge about infectious diseases, including behavior tips, treatment methods, and transmission routes. Such programs mitigate the adverse effects of the COVID-19 infodemic, balancing positive and negative consequences. The significance of this study is underscored by the identification of the asymmetry in COVID-19 information among healthcare professionals working in primary, secondary, and tertiary hospitals, which implies the need for future research to explore and measure the concept of asymmetry of COVID-19 information among these healthcare professionals.

## Data availability statement

The original contributions presented in the study are included in the article/supplementary material, further inquiries can be directed to the corresponding author.

## Ethics statement

The studies involving humans were approved by the Soonchunhyang University Institutional review board (1040875-202307-SB-077). The studies were conducted in accordance with the local legislation and institutional requirements. The participants provided their written informed consent to participate in this study.

## Author contributions

SC: Conceptualization, Data curation, Formal analysis, Funding acquisition, Investigation, Methodology, Project administration, Resources, Software, Supervision, Validation, Visualization, Writing – original draft, Writing – review & editing.
